# Associations of stress and stress-related psychiatric disorders with GrimAge acceleration: review and suggestions for future work

**DOI:** 10.1038/s41398-023-02360-2

**Published:** 2023-05-02

**Authors:** Ekaterina Protsenko, Owen M. Wolkowitz, Kristine Yaffe

**Affiliations:** 1 Department of Psychiatry, Stanford University School of Medicine, Palo Alto, CA USA; 2 Department Epidemiology & Biostatistics, University of California San Francisco (UCSF) School of Medicine, San Francisco, CA USA; 3 Weill Institute for Neurosciences and Department of Psychiatry and Behavioral Sciences, University of California San Francisco (UCSF) School of Medicine, San Francisco, CA USA; 4 Department of Neurology, University of California San Francisco (UCSF) School of Medicine, San Francisco, CA USA

**Keywords:** Neuroscience, Predictive markers

## Abstract

The notion of “biological aging” as distinct from chronological aging has been of increasing interest in psychiatry, and many studies have explored associations of stress and psychiatric illness with accelerated biological aging. The “epigenetic clocks” are one avenue of this research, wherein “biological age” is estimated using DNA methylation data from specific CpG dinucleotide sites within the human genome. Many iterations of the epigenetic clocks have been developed, but the GrimAge clock continues to stand out for its ability to predict morbidity and mortality. Several studies have now explored associations of stress, PTSD, and MDD with GrimAge acceleration (GrimAA). While stress, PTSD, and MDD are distinct psychiatric entities, they may share common mechanisms underlying accelerated biological aging. Yet, no one has offered a review of the evidence on associations of stress and stress-related psychopathology with GrimAA. In this review, we identify nine publications on associations of stress, PTSD, and MDD with GrimAA. We find that results are mixed both within and across each of these exposures. However, we also find that analytic methods — and specifically, the choice of covariates — vary widely between studies. To address this, we draw upon popular methods from the field of clinical epidemiology to offer (1) a systematic framework for covariate selection, and (2) an approach to results reporting that facilitates analytic consensus. Although covariate selection will differ by the research question, we encourage researchers to consider adjustment for tobacco, alcohol use, physical activity, race, sex, adult socioeconomic status, medical comorbidity, and blood cell composition.

## Introduction

In recent decades, there has been mounting scientific evidence in support of the long-held idea that psychological stress contributes to accelerated aging. Colloquially, this “accelerated aging” is understood as the general sense that people of the same chronological age can differ in their physical appearance, fitness, and functioning. Scientifically, accelerated biological aging has been defined through many models, including allostatic load [1], telomere length [2, 3], and most recently, epigenetic age [4], among others. These approaches differ in their ability to predict future morbidity and mortality, and it is in this respect that the so-called “epigenetic clocks” represent a significant advancement over their predecessors [5, 6].

The epigenetic clocks are based on the finding that chronological age has predictable effects on DNA methylation at a subset of the genome’s 28 million CpG dinucleotide sites [4]. Although several epigenetic clocks have been developed, their underlying premise is the same. Each clock consists of a specific set of CpG dinucleotide sites and an accompanying algorithm that together output an estimate of epigenetic age. When epigenetic age exceeds an individual’s chronological age, they experience “epigenetic age acceleration” (EAA). Conversely, when an individual’s epigenetic age is less than their chronological age, they experience epigenetic age deceleration. Formally, EAA is defined as the residual resulting from regressing epigenetic age on chronological age.

While the underlying premise of the various epigenetic clocks is the same, they differ with respect to the target of prediction during their derivation. The first-generation epigenetic clocks — i.e., Horvath [7] and Hannum [8] clocks — were derived by machine learning to predict an individual’s chronological age. In other words, machine learning was used to identify those CpG sites whose methylation states were most predictive of true chronological age. However, one of the pitfalls of this approach was that it systematically excluded CpG sites whose methylation states signaled a departure from the normal trajectory of aging [9]. This issue in turn gave rise to the second-generation of epigenetic clocks, known as PhenoAge [9] and GrimAge [10], which were derived to predict clinical phenotype and time-to-death, respectively, rather than chronological age. Consistent with their derivation, both PhenoAge and GrimAge outperformed the first-generation clocks in their ability to predict morbidity and mortality [9, 10]. Other epigenetic clocks include the DunedinPOAM [11] and DunedinPACE [12] clocks derived from a longitudinal cohort in New Zealand, as well as several clocks for special populations [13–15], and the field continues to grow.

Since their description, the epigenetic clocks have garnered significant interest in clinical research because they offer a means to consider the implications of a wide variety of exposures for disease and lifespan when traditional longitudinal data may not be available. The use of an epigenetic proxy for morbidity and mortality brings with it caveats, and authors often note that it remains unknown if findings of excess EAA correspond to a true excess burden of morbidity or mortality for any given exposure. Yet there are other complexities to causal inference with the epigenetic clocks that are much less commonly discussed in research reports. One approach to framing this complexity is to consider that the epigenetic clocks are the products of research with *predictive aims* that are being applied to research with *causal inference aims*. In other words, the research aim during the derivation of the clocks was to create the most accurate tool possible for predicting the target (e.g., time-to-death) while remaining agnostic to the mechanistic relevance of the CpG sites included. Some of the CpG sites may be part of cellular pathways directly related to disease and death, while others may be proxies for biologically relevant health-related behaviors (e.g., tobacco use) but have no mechanistic relevance themselves. Researchers then use this tool as an outcome measure for research with causal inference aims, e.g., to understand whether a given exposure—such as MDD—might be a causal contributor to increased EAA. Adequately framing questions about the causal relationship between a psychiatric exposure and morbidity or mortality requires a great deal of nuance. Research questions about the causal relationship between a psychiatric exposure and epigenetic age acceleration require the same considerations. However, unlike with morbidity and mortality, there is still only limited information available on the confounders, mediators, and effect modifiers that may be relevant to the study of the epigenetic clocks. As a result, even simple research questions about EAA are rife with uncertainty.

Still more complexity is introduced when one considers the unique properties of each specific epigenetic clock, most notably so with GrimAge. Unlike the earliest iterations of the epigenetic clocks, where, the target of prediction was simply chronological age, the GrimAge clock was derived by a complex two-stage approach in order to predict time-to-death [10]. In the first stage, the authors began with 88 plasma proteins whose levels have been previously associated with mortality. For each of these 88 candidate biomarkers, they derived a DNAm-based surrogate for the plasma protein. (Of the 88 plasma proteins considered, DNAm-based surrogates were successfully created for only 12.) The same process was used to derive a DNAm-based surrogate for self-reported lifetime smoking exposure. In the second stage, these 13 DNAm-based surrogates, as well as chronological age and sex, were used to derive an algorithm to predict time-to-death. The resulting GrimAge Clock consisted of 8 of the original 12 DNAm surrogates for plasma proteins, the DNAm surrogate for smoking exposure, and age and sex, accompanied by an algorithm that transformed estimates of time-to-death into the now familiar form of epigenetic age (for more, see Lu et al. [10]). As a result of this derivation, variables that might be considered necessary covariates in a study of the association of psychiatric illness with morbidity or mortality — such as smoking exposure — are now part-and-parcel of the epigenetic clock itself. In turn, this tool that excels at its stated goal of predicting lifespan and healthspan presents obstacles for covariate selection when used in causal inference research.

Despite these challenges, the epigenetic clocks have also garnered interest in the field of psychiatry. The relevance of the epigenetic clocks to psychological stress was first demonstrated by Zannas et al. in 2015, when they reported that cumulative lifetime stress was associated with greater EEA when measured with the Horvath clock [16]. However, later studies examining stress [12–14], post-traumatic stress disorder (PTSD) [17–23], and major depressive disorder (MDD) [24–28] with the epigenetic clocks have shown mixed results. Despite this growing body of conflicting findings, there has not been a systematic review of epigenetic age in stress and stress-related psychopathology. In this review, we summarize the evidence on the association of psychosocial stress, PTSD, and MDD with GrimAge, and consider how methodological challenges related to the epigenetic clocks may contribute to mixed results. We limit our review to GrimAge in order to facilitate discussion of analytic methods for one of the most methodologically complex epigenetic clocks available, and because there is an adequate number of published studies for comparison. Of note, our focus is on describing principles and frameworks that may be applied to the other epigenetic clocks, rather than on offering concrete recommendations to be employed in all studies of epigenetic age.

## Methods

### Data sources and search strategy

**Fig. 1:**
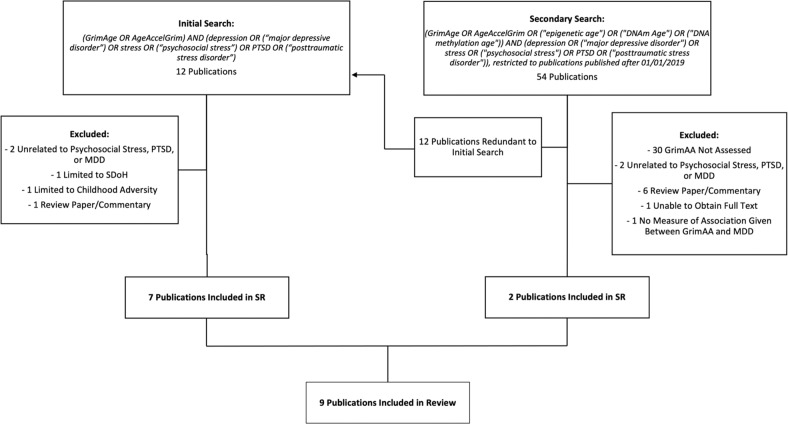
Search strategy and study selection. All searches were completed on 03/08/2022 in Medline. SDoH social determinants of health.

We searched Medline on March 8, 2022 for the terms: *(GrimAge OR AgeAccelGrim) AND (depression OR (“major depressive disorder”) OR stress OR (“psychosocial stress”) OR PTSD OR (“posttraumatic stress disorder”))*. Due to concern that the narrow search term “*(GrimAge OR AgeAccelGrim)*” might fail to yield all relevant publications, we completed a second broader search also on March 8, 2022 using: *(GrimAge OR AgeAccelGrim OR (“epigenetic age”) OR (“DNAm Age”) OR (“DNA methylation age”))*. This search was restricted to publications after 01/01/2019 to exclude any papers prior to the publication of the GrimAge clock (Fig. 1).

### Study selection

All titles and abstracts were reviewed by one author (EP). Review papers and studies where GrimAge was not measured were excluded. Studies that did not assess psychosocial stress, PTSD diagnosis or symptoms, or MDD diagnosis or symptoms were also excluded. For any studies where it was not clear from the abstract whether GrimAge—as opposed to another epigenetic clock—was measured, the full text was reviewed. Studies were included if they provided a direct measure of association between GrimAge acceleration (GrimAA) and a validated and or standardized metric of psychosocial stress, PTSD/MDD diagnosis, or PTSD/MDD symptom severity. Studies limited to childhood adversity or social determinants of health were considered beyond the scope of this review.

### Data extraction and synthesis

For all papers selected for further review, full texts were obtained and reviewed by EP. Data were extracted on study sample and size, study design, instruments used, tissue assayed, covariates, and relevant main findings (Table 1). All data were extracted by EP. For the purposes of this review, stress/stress-related psychopathology was considered the exposure (independent variable) and GrimAA was the outcome (dependent variable). Questions or concerns about data extraction were addressed by consulting with other authors.

**Table 1:** Summary of the literature.

Study	Sample Size and Description	Study Design	Exposure	GrimAge Measured in	Covariates	Main Findings
Stress						
Harvanak et al., 2021	*N* = 444 Mean age: 28.6 (SD = 8.7) 44.8% Male Restricted to somatically healthy adults free of dependence on any drugs other than alcohol or nicotine, and not using medications for any psychiatric condition. Convenience community sample in New Haven, CT, recruited by local advertising	Cross-sectional	- Cumulative stress: Cumulative Adversity Index (CAI)	Whole blood	- Sex - Race - Years of education - Marital status - Income - Smoking (also considered as mediator) - Alcohol use (also considered as mediator) - BMI (also considered as mediator) - Blood cell composition (Houseman estimates, supplementary analysis)	Greater cumulative stress (total CAI score) is associated with greater GrimAA ***(Unadjusted: t = 4.82, P = 2.00e−6, η***^***2***^ ***= 0.050, adjusted R***^***2***^ ***= 0.0478)*** ***(Adjusted: t = 2.073, P = 0.0388, partial η***^***2***^ ***= 0.010)*** ***(Adjusted w/ Houseman estimates: coef: 0.0028, P=0.0099)*** Effect of stress on GrimAA may be partially mediated by BMI and insulin resistance ***(Indirect effect via BMI and HOMA: coef: 0.003, P*** ***=*** ***0.030*** ***Direct effect: coef: 0.034, P*** ***=*** ***0.009)***
McKenna et al., 2021	*N* = 219 Mean age: 45.9 (SD = 12.3) 36.1% Male Restricted to Black participants. Sub-sample of Grady Trauma Project, an inner-city primary care sample of >10,000 individuals recruited from primary care and ObGyn waiting rooms at a single large public hospital in Atlanta, GA. (238 individuals completed who provided blood and completed additional questionnaires; 19 non-Black participants excluded)	Cross-sectional	- Lifetime trauma exposure: Traumatic Events Inventory (TEI) Stressful life events: Stressful Events Questionnaire I - Race-related lifetime stress exposure (LSE): a composite score, calculated as average of standardized scores on Experiences of Discrimination survey, Stressful Events Questionnaire I, and Traumatic Events Inventory	Whole blood	- Income - Sex - Blood cell composition (Houseman estimates)	Race-related lifetime stress exposure not associated with GrimAA ***(Unadjusted: R = 0.04, P = 0.59)*** ***(Adjusted: ∆F = 0.23, ∆R***^***2***^ ***= 0.001, t = 0.48, P = 0.64)*** Similarly, neither lifetime trauma exposure (TEI score) nor stressful life events (Stressful Events Questionnaire I) are associated with GrimAA ***(Trauma Exposure: R = 0.06, P = 0.37)*** ***(Stressful Life Events: R = −0.04, P = 0.55)***
Post-Traumatic Stress Disorder						
Katrinli et al., 2020	*Discovery:* *N* = 854 218 Current PTSD: Mean age: 40.9 (SD = 11.4) 20.2% Male 209 Prior PTSD: Mean age: 43.3 (SD = 11.8) 34% Male 427 PTSD-free controls: Mean age: 42.8 (SD = 12.7) 33.9% Male Restricted to those reporting ≥1 traumatic event Grady Trauma Project - Atlanta, GA	Cross-sectional with exposure-selective sampling	- Lifetime trauma exposure: Traumatic Events Inventory (TEI) - PTSD diagnosis: Clinician-Administered PTSD Scale (CAPS) *or* Modified PTSD Symptomatic Scale (mPSS) if missing CAPS data (12.8% of sample)	Whole blood	- Sex - Array type (MethylationEPIC vs Methylation450 BeadChip by Illumina) - Blood cell composition (Houseman estimates)	In adjusted linear regressions, both current PTSD and any history of lifetime PTSD are associated with excess GrimAA compared to PTSD-free controls ***(Current PTSD v. PTSD-free control: ß = 0.86, SE = 0.38, t = 2.25, P = 0.02)*** ***(any history of lifetime PTSD v. PTSD-free control: ß = 0.87, SE = 0.31, t = 2.80, P = 0.005)*** In post hoc pairwise comparisons, GrimAA did not differ between current PTSD and prior PTSD ***(P = 0.95)***, suggesting that effects of PTSD on GrimAA may not be reversible. Greater Lifetime Trauma Exposure (TEI score) is associated with greater GrimAA ***(Adjusted: ß = 0.12, SE = 0.06, t = 2.13, P = 0.03)***
	*Replication:* *N* = 309 120 Lifetime PTSD: Mean age: 53.0 (SE = 13.7) 32.5% Male 189 PTSD-free controls: Mean age: 56.6 (SE = 13.5) 40.2% Male Subcohort of Detroit Neighborhood Health Study, for whom GrimAA data were available	Cross-sectional with exposure-selective sampling	- Trauma exposure: number of traumatic event types experienced (see Breslau et al.) - PTSD diagnosis: PTSD Checklist Civilian Version (PCL-C)	Whole blood	- Sex - Array type (MethylationEPIC vs Methylation450 BeadChip by Illumina) - Blood cell composiiton (Houseman estimates)	Lifetime PTSD is associated with excess GrimAA compared to PTSD-free controls ***(Adjusted: ß = 1.38, SE = 0.67, t = 2.04, P = 0.04)*** The association between Lifetime Trauma Exposure was in the same direction as in the Discovery cohort, but not significant. ***(Adjusted: ß = 0.13, SE = 0.07, t = 1.87, P = 0.06)***
Mehta et al., 2021	*N* = 39 Mean age = 23.4 (SE = 1.1) 38.5% Male First-year undergraduate paramedicine students at Australian University	Longitudinal pre-post paramedicine fieldwork exposure	- PTSD symptom severity: PTSD Checklist for DSM-5 (PCL-5) - Psychological Distress: Kessler K10 Scale	Saliva	- Age - Ethnicity - BMI - Alcohol - Smoking - Blood cell composition (Middleton method) - Medication	GrimAA measured prior to fieldwork exposure (pre-stress exposure) is not associated with PTSD symptom severity either at baseline or follow-up ***(p > 0.05)*** GrimAA measured after fieldwork exposure (post-stress exposure) is associated with PTSD symptom severity both at baseline and follow-up ***(post-stress GrimAA v. pre-stress PTSD Symptoms: R = 0.39, P = 0.0091)*** ***(post-stress GrimAA v. post-stress PTSD symptoms: R = 038, P = 0.0084)*** Psychological distress is not associated with GrimAA either pre- or post-fieldwork exposure ***(p > 0.05)***
Kuan et al., 2021	*N* = 324 Mean age = 51.8 (SD = 8.1) 100% Male 81 Current PTSD, 42 past PTSD, 201 no PTSD history Previously assembled cohort of World Trade Center (WTC) responders from the Stony Brook World Trade Center Health Program	Cross-sectional with exposure-selective sampling	- PTSD diagnosis: Structured Clinical Interview for DSM-IV (SCID) - PTSD symptom severity: PTSD Checklist - Specific Version (PCL-17) for severity of WTC-related PTSD symptoms	Whole blood	- Age - Race - Blood cell composition (Houseman estimates, supplementary sensitivity analysis)	Compared to WTC responders with no history of PTSD, those with current PTSD showed excess GrimAA ***(Adjusted: ß = 0.34, P = 0.010)*** ***(Adjusted with Blood Cell Composition: ß = 0.171, P = 0.002)*** GrimAA did not differ between current and past PTSD, or between past PTSD and no PTSD ***(Current v. past PTSD: ß = 0.19, P = 0.386)*** ***(past PTSD v. never PTSD: ß = 0.132, P = 0.410)*** GrimAA is positively associated with total PTSD severity, as well as with the avoidance and numbing symptom domains, but not with re-experiencing or hyperarousal ***(0.05 < P < 0.1)*** ***(Total PTSD Severity: ß = 0.146, P = 0.010)*** ***(Avoidance: ß = 0.147, P = 0.0083)*** ***(Numbing: ß = 0.186, P = 0.012)***
Yang et al., 2021	*Discovery:* *N* = 162 80 Combat trauma-exposed veterans with PTSD: Mean age: 32.7 (SD = 7.4) 100% Male 82 PTSD-free combat trauma exposed veteran controls: *Mean age: 32.5 (SD = 8.0)* *100% Male* *Replication:* *N* = 53 26 PTSD-positive: Mean age: 36.9 (SD = 10.2) 100% Male 27 PTSD-free controls: Mean age: 34.0 (SD = 9.4) 100% Male Combat veterans from Operation Enduring Freedom and/or Operation Iraqi Freedom, free of current or recent drug or alcohol dependence, recent or ongoing traumatic experiences, and any illness affecting the CNS, and with stable medication regimens	Cross-sectional with exposure-selective sampling	- PTSD diagnosis: SCID - PTSD symptom severity: Clinician Administered PTSD Scale (CAPS)	Whole blood	- BMI - CD8+CD28− cell proportion (flow cytometry) - Smoking (plasma cotinine levels) - Blood cell composition (Houseman estimates)	Veterans with PTSD have greater GrimAA compared to the veterans without PTSD ***(Discovery unadjusted: 1.26 ± 3.93 years GrimAA in PTSD v. −0.57 ± 3.38 years GrimAA in control, t = −3.184, P = 0.001)*** ***(replication unadjusted: 0.93 ± 3.73 years GrimAA in PTSD v. −1.60 ± 2.96 years GrimAA in PTSD-, t = −2.725, P = 0.008)*** ***(combined adjusted (N = 175): t = 2.25, P = 0.026; attenuated to P = 0.056 when using self-report smoking instead of cotinine but the N = 175 due to missing data)*** PTSD symptom severity is associated with GrimAA across all participants ***(Across cohorts: r = 0.236, P < 0.001)*** Three-year follow-up of PTSD subcohort (*N* = 26, with PTSD at baseline): Longitudinal changes in GrimAA are positively correlated with longitudinal changes in PTSD symptom severity ***(r = 0.391, P = 0.049)***
Wang et al., 2022	*N* = 296 89 Monozygotic pairs, 44 dizygotic pairs, 30 individuals without twin member. 21 Twin pairs discordant with respect to PTSD status. 24 Participants with current PTSD: Mean age: 57.4 (SD = 2.2) 100% Male 272 Participants with no current PTSD: Mean age: 56.0 (SD = 3.4) 100% Male Subgroup of Vietnam Era Twin Registry, participating in Emory Twin Study	Cross-sectional sampling of twin pairs	- PTSD diagnosis: Structured Clinical Interview for DSM-IV (SCID) - PTSD symptom severity: Clinician Administered PTSD Scale (CAPS) (*N* = 183)	PBMCs	- Smoking - BMI - Alcohol Consumption - Baecke Score for Physical Activity *Sensitivity analyses:* - Comorbid major depressive disorder (Model 2) - Antidepressant use (Model 3) - Combat Exposure Scale Score (Model 4)	In linear mixed models adjusting for co-twins as repeated measures, GrimAA does not differ between individuals with current PTSD and without current PTSD ***(unadjusted mean difference current PTSD v. no PTSD: 1.63 years GrimAA (95% CI = −0.65 to 3.91), p = 0.16)*** GrimAA remained unassociated with PTSD status in adjusted models (twins treated as individuals). ***(Adjusted, Model 1: ß = 1.54 (−0.17 to 3.24), P = 0.079)*** ***(Adjusted, Model 2: ß = 1.38 (−0.37 to 3.12), P = 0.12)*** ***(Adjusted, Model 3: ß = 1.67 (−0.12 to 3.45), P = 0.069)*** ***(Adjusted, Model 4: ß = 0.58 (−1.33 to 2.49), P = 0.55)*** In models restricted to twin pairs discordant with respect to PTSD status in paired *t* test, GrimAA did not differ between those with current PTSD and those without current PTSD ***(unadjusted mean diff current PTSD v. no PTSD: 1.46 years GrimAA (95% CI = −1.24 to 4.16), P = 0.27)*** In models considering twins as individuals, GrimAA was not associated with PTSD Symptom severity ***(Adjusted, Model 1: ß = 0.001 (−0.034 to 0.036), P = 0.96)*** ***(Adjusted, Model 2: ß = −0.026 (−0.039 to 0.034), P = 0.89)*** ***(Adjusted, Model 3: ß = −0.001 (−0.039 to 0.036), P = 0.94)*** ***(Adjusted, Model 4: ß = −0.010 (−0.052 to 0.032), P = 0.63)***
Major Depressive Disorder						
Protsenko et al., 2021	*N* = 113 50 Participants with moderate-to-severe depression: Mean age: 40.2 (SD = 14.6) 45% Male 63 Depression-free controls: Mean age: 39.4 (SD = 13.6) 40% Male restricted to somatically healthy adults San Francisco, CA clinic and community sample	Cross-sectional with exposure-selective sampling	- Depression diagnosis: Structured Clinical Interview for DSM-IV TR (SCID) - Depression symptom severity among MDD: Hamilton Depression Rating Scale (HDRS) - Depression symptom severity across all participants: Inventory of Depressive Symptoms (IDS-SR)	Whole blood	- Current smoking status (lifetime smoking status in sensitivity analysis) - Sex - BMI - Blood cell composition (supplementary analyses, CBC values)	Patients with MDD demonstrate excess GrimAA compared to MDD-free controls ***(MDD, median GrimAA ± IQR: 0.36 years ± 2.18 years)*** ***(HC, median GrimAA ± IQR: −1.64 years ± 3.95 years)*** ***(MDD v. HC, unadjusted: t = −3.27, P = 0.001, Cohen’s d = 0.6)*** ***(MDD v. HC, adjusted: F = 6.1, P = 0.02)*** Among participants with MDD, GrimAA is not associated with scores of depression severity on the HDRS, lifetime days of depression, days of untreated depression, chronicity of lifetime depression, or Duration of the current depressive episode. ***(GrimAA v. HDRS: r = −0.09, P = 0.55)*** ***(GrimAA v. Lifetime days of depression: r = −0.01, P = 0.95)*** ***(GrimAA v. days of untreated depression: r = 0.06, P = 0.68)*** ***(GrimAA v. chronicity of depression: r = −0.01, P = 0.93)*** ***(GrimAA v. duration of current depressive episode: r = −0.13, P = 0.39)***
Vetter et al., 2022	*N* = 1100 Mean age: 75.60 (SD = 3.8) 47.9% Male Convenience sample of older adults aged 60–85 from the Greater Metropolitan Area of Berlin, Germany, and who returned for follow-up on average 7.4 years later	Cross-sectional at cohort follow-up	- Depression symptom severity: Center for Epidemiologic Studies - Depression Scale (CES-D)	Not specified	- Genetic ancestry (principal components analysis of genome-wide SNP data) - Age - Sex - Alcohol Consumption - Smoking - Morbidity Index (Modified Charlson Index) - BMI	GrimAA is not associated with CES-D scores of depression severity ***(age and sex adjusted (N = 1041): ß = −0.01, SE = 0.04, P = 0.63)*** ***(fully adjusted (N = 789): ß = 0.01, SE = 0.04, P = 0.81)***

*GrimAA* GrimAge acceleration, *PTSD* post-traumatic stress disorder.

## Results

### Description of search yield

The initial search yielded 12 publications in Medline (Fig. 1). Of these, 7 met criteria for inclusion. The broader search term yielded 54 publications. As expected, this included the same 12 publications. Of the remaining 42, only 2 met criteria for inclusion. The nine included publications are summarized in Table 1.

### Study characteristics

Of the nine studies, two examined the association between psychosocial stress and GrimAA, five between PTSD and GrimAA, and two between depression and GrimAA. Seven studies were cross-sectional. Of these, three [29–31] studies used community-based cross-sectional sampling, three used exposure-selective sampling (i.e., diagnosis of MDD or PTSD is the exposure) [28, 32, 33], and one was a twin study where pairs of twins were selected on the basis of PTSD diagnosis [34]. The eighth study was a longitudinal pre-post stress exposure [35]. The ninth study was described as a case-control investigation of PTSD [36]. However, because we defined psychiatric diagnosis as the exposure and GrimAA as the outcome, this study was reframed as exposure-selective cross-sectional sampling.

Sample sizes ranged from 39 to 1100. Study populations included community-based urban samples, paramedicine students, first-responders, combat veterans, twins from the Vietnam Era Twin Registry, and matched clinic and community samples with and without MDD. Two studies restricted inclusion to somatically healthy adults [28, 30], and one study excluded individuals with any disorder affecting the central nervous system [33]. Three studies were restricted to males [33, 34, 36]. Mean sample age ranged from 23.4 to 78.6 years, although most studies were conducted in mid-life adults. Mehta et al. studied a younger sample of undergraduates, while Vetter et al. studied older adults. Three studies stood out for limited variability in chronological age: Mehta et al. (mean age = 23.4, SD = 1.1), Vetter et al. (mean age = 75.6, SD = 3.8), and Wang et al. (mean age = 57.4, SD = 2.2). Most studies were conducted in the USA, except one in Australia and one in Germany. Of note, two studies were conducted in sub-samples of the same parent cohort: Katrinli et al. and McKenna et al. both examined subsets of the Grady Trauma Project.

### Psychosocial stress and GrimAA

Two studies assessed associations between lifetime psychosocial stress and GrimAA in cross-sectional samples of urban adults, with inconsistent results. Harvanek et al. found that greater cumulative stress measured by the cumulative adversity index [37] was associated with greater GrimAA (*p* = 2.0e−6) and that this association persisted after extensive adjustment for behavioral and demographic determinants of health (*p* = 0.01), as well as blood cell composition (*p* = 0.01). However, McKenna et al. found no evidence of an association between GrimAA and lifetime trauma [38] (*p* = 0.37), number of stressful life events [39] (*p* = 0.55), or race-related lifetime stress exposure [40] (LSE; *p* = 0.59). However, they did find evidence of an indirect association between race-related lifetime stress exposure and GrimAA, mediated by internalized anger. While both studies were completed in community-based urban samples, the McKenna et al. sample was on average older, not restricted to somatically healthy individuals, and recruited from outpatient healthcare settings rather than from the general community. The exposure was also defined using different instruments, including interviews and questionnaires. Notably, although findings were significant in Harvanek et al., effect sizes were small, with *η*^2^ equal to 0.05 in unadjusted and 0.01 in adjusted models.

One additional study, Mehta et al. assessed GrimAA in a sample of undergraduate students before and after their first paramedicine fieldwork experience. They found no evidence of association between psychological distress and GrimAA in the past 30 days [41], either pre- or post-fieldwork exposure (*p* > 0.05). Unlike the prior studies, Mehta et al. assessed only recent subjective distress rather than cumulative lifetime stress.

### Post-traumatic stress disorder and GrimAA

Five studies examined the associations between GrimAA and PTSD in civilian, first responder, and military samples. Katrinli et al. found that highly traumatized urban civilians without PTSD from the Grady Trauma Project had lower GrimAA than those with current or prior PTSD. Compared to their PTSD-free counterparts, those with current PTSD had on average 0.86 years of excess GrimAA (*p* = 0.02), while those with any lifetime history of PTSD (i.e., current or prior) had 0.87 years of excess GrimAA (*p* = 0.005). Across PTSD groups, greater lifetime trauma exposure was associated with greater GrimAA, with 0.12 years of excess GrimAA per one unit increase on the Traumatic Events Inventory (*p* = 0.03). A similar pattern of findings was observed in a replication from the Detroit Neighborhood Health Study (Table 1).

Mehta et al. also assessed GrimAA and PTSD symptoms in a civilian sample of undergraduates pre- and post- paramedicine fieldwork exposure, as described above. In contrast, GrimAA measured at baseline was not associated with baseline PTSD symptom severity, nor did it predict PTSD symptoms after fieldwork exposure (*p* > 0.05). However, GrimAA measured after fieldwork exposure was correlated with PTSD symptom severity both pre-fieldwork (*p* = 0.009) and post-fieldwork (*p* = 0.008).

Among World Trade Center first-responders, Kuan et al. reported that those with current PTSD had excess GrimAA compared to first-responders with no history of PTSD (*p* = 0.01), with on average 0.34 years of excess aging. Across PTSD groups, GrimAA was associated with PTSD symptom severity (*p* = 0.01).

Two studies examined the association between GrimAA and PTSD among military veterans. Yang et al. (2021) studied 215 young male veterans who had been exposed to combat trauma. Those with PTSD had greater GrimAA than their PTSD-free combat-exposed counterparts in two separate cohorts, with 1.8 and 5.5 years of excess GrimAA in unadjusted models, respectively. Across PTSD groups, PTSD symptom severity was associated with GrimAA (*p* < 0.001). Wang et al. studied GrimAA in a cohort of older men from the Vietnam Era Twin Registry. When co-twins were treated as repeat measures, GrimAA did not differ between individuals with and without PTSD (*p* = 0.16). Similarly, no differences were identified when the analysis was restricted to twin pairs discordant for PTSD status (*p* = 0.27), nor was GrimAA associated with PTSD symptom severity across the full sample (*p* > 0.5 in serially adjusted models).

### Major depressive disorder and GrimAA

Two studies examined the association of GrimAA with depression. Our group previously demonstrated that, in a sample of somatically healthy patients with moderate-to-severe untreated depression and depression-free controls, those with depression had a median two years of excess GrimAA compared to controls (unadjusted *p* = 0.001, adjusted *p* = 0.02). However, among patients with MDD, GrimAA was not correlated with depression severity, duration of depression, or duration of the current depressive episode [28]. Vetter et al. (2022) assessed GrimAA cross-sectionally in a large sample of older adults from Berlin, Germany. Depressive symptom severity was not associated with GrimAA (*p* > 0.5). In contrast to Protsenko et al., Vetter et al. assessed depressive symptoms in a population-based sample rather than a clinical sample of depressed patients. Vetter et al. is also the only study of the nine reviewed to assess GrimAA in a geriatric sample (mean age = 75.6 years), with limited variability in age.

### Comparing study covariates

With the exception of Mehta et al. and studies that were restricted to males, all studies adjusted for sex. Only six studies adjusted for BMI, six for smoking status, four for alcohol consumption, and five for race/ethnicity/genetic ancestry (McKenna et al. by restriction to Black participants). Only two studies adjusted for socioeconomic status by adjusting for years of education and/or income. Only one study adjusted for physical activity. Seven of the nine studies adjusted for blood cell composition. Of these, 5 used the Houseman estimates of blood cell composition based on DNAm data [42] (http://dnamage.genetics.ucla.edu). One study used complete blood counts, while another used the Middleton method [43].

### Effect sizes

Effect sizes were generally small-to-moderate. Of the studies comparing GrimAA between those with and without PTSD, two studies found that PTSD was associated with less than one year of excess GrimAA (0.34 years in Kuan et al., 0.86–0.87 years in Katrinli et al.). However, Yang et al. observed larger effect sizes, with veterans with PTSD exhibiting 1.8 years of excess GrimAA in the discovery cohort, and 5.5 years of excess GrimAA in the smaller replication cohort. As noted before, Yang et al. is the only study of PTSD to restrict inclusion to somatically healthy individuals. Wang et al. also observed 1.63 years of excess GrimAA among those with PTSD compared to those without, but this result was not statistically significant (95% CI −0.65 to 3.91 years of GrimAA). Only one study compared GrimAA between individuals with and without MDD, and found that MDD was associated with a median 2 years of excess aging (Protsenko et al.).

Two studies reported standardized measures of effect size. Harvanek et al. reported that while the association between stress and GrimAA was statistically significant, effect sizes based on *η*^2^ were small (*η*^2^ = 0.05 in unadjusted and 0.01 in adjusted models). Protsenko et al. reported a Cohen’s *d* of 0.6 for unadjusted comparisons of patients with MDD to healthy MDD-free controls. For all studies providing sufficient information to do so, Cohen’s *d* values were calculated (Supplementary Table 1). Cohen’s *d* ranged from 0.16 to 0.75, consistent with small-to-moderate effects.

## Discussion

In this review, we considered the body of evidence on the association between stress and stress-related psychopathology with acceleration of the GrimAge epigenetic clock. We identified nine original research publications, and found that five of these — Harvanek et al., Katrinli et al., Kuan et al., Yang et al., and Protsenko et al. — reported statistically significant associations between GrimAA and stress, MDD, and/or PTSD. Three studies — McKenna et al., Wang et al., and Vetter et al. — found no association, and Mehta et al. reported mixed results. PTSD was the most well-studied exposure and had the strongest evidence in support of an association with GrimAA, although conflicting findings nonetheless prevent a clear conclusion. As the literature grows, we take this opportunity to consider how differences in methodology specific to the study of epigenetic age in psychiatric illness may contribute to conflicting results. We apply a theoretical framework from the field of epidemiology as both a conceptual model and practical tool.

**Fig. 2:**
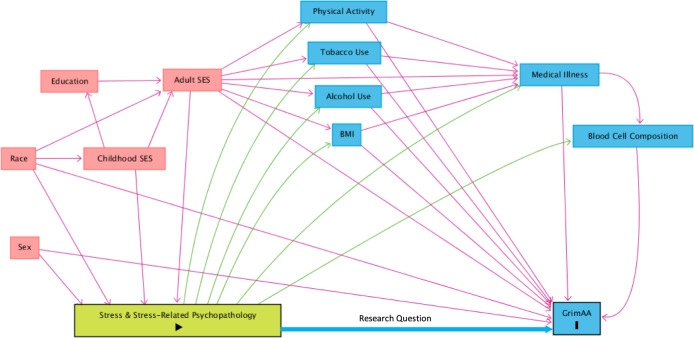
Directed acyclic graph depicting the relationships between stress and stress-related psychopathology and GrimAA. Green lines indicate causal or mediating paths. Pink lines indicate confounding paths. Minimally sufficient adjustment set (MSAS) for direct effect of stress and stress-related psychopathology on GrimAA = Tobacco Use + Alcohol Use + BMI + Physical Activity + Medical Illness + Blood Cell Composition + Sex + Race + Adult SES. MSAS for Total Effect = Adult SES + Race + Sex. Generated with daggity.net, modified for clarity. Supplementary Materials include code to reconstruct this DAG at dagitty.net.

As noted previously, the nine studies considered in this review differed in their choice of covariates, study samples, and study designs. While study samples and designs will naturally vary, the lack of consensus as to covariates suggests a lack of consensus in general regarding analytic best practices and the precise research question. In order to facilitate consensus, the field of epidemiology has developed a rigorous tool known as the directed acyclic graph (DAG) [44]. DAGs encourage the researcher to shift focus from individual covariates to imagining a complex network of variables that represent the broader biopsychosocial system within which the research question exists. Once a DAG has been constructed, a number of essential points become clear, such as the distinction between direct and indirect causal effects and the risks of overadjustment. Analysis covariates are then chosen a priori on theoretical grounds. In Fig. 2, we provide one depiction of the system surrounding the relationship of stress, MDD, and PTSD with GrimAA using a DAG. Due to still limited literature on GrimAA, Fig. 2 should be considered a preliminary attempt at consensus that builds upon prior work identifying relevant covariates [45–48] (for a review, see Oblak et al. [45]). It should also be noted that DAGs will differ for different research questions, and Fig. 2 should be regarded as a starting point. Supplementary Fig. 1 details how to construct this DAG and its rationale. As knowledge about the epigenetic clocks grows, the DAG will also evolve and may perhaps include such variables as air pollution [49], sleep [50], and diet [51], among others.

As Fig. 2 demonstrates, the causal effect of stress and psychopathology on GrimAA may be confounded and mediated by many variables, and these variables in turn relate to one another in complex ways. Beginning with mediation, the effect of stress and psychopathology on GrimAA is at least in part mediated by behavioral variables like tobacco and alcohol use, and by comorbid somatic illness. Measures of association that do not adjust for these variables provide the “total causal effect.” However, the primary research question in this review is about the biological rather than behavioral processes, i.e., about the “direct causal effect.” In other words, researchers ask whether stress and stress-related psychopathology contribute to GrimAA independent of their effects on health-related behaviors, perhaps through mechanisms like autonomic, neuroendocrine, immune, and other biological changes [52–54]. Generating informative measures of association of direct causal effects therefore requires controlling for tobacco use, alcohol use, physical activity, and BMI, either by statistical adjustment, sample restriction, or other means. However, of the five publications reporting significant findings, only one (Harvanek et al.) accounted for alcohol use, tobacco use, and BMI. Protsenko et al. and Yang et al. adjusted only for BMI and tobacco use, while Katrinli et al. and Kuan et al. did not adjust for any of the three suspected behavioral mediators. In contrast, of the four remaining studies reporting no significant associations or equivocal results, one study adjusted for all four behavioral mediators, and two others for three mediators. It is not clear if the associations in Protsenko et al., Yang et al., Katrinli et al., and Kuan et al. would persist if behavioral mediators were further accounted for.

Additionally, there is one health-related behavior that warrants particular attention in the discussion of GrimAge: tobacco use. As noted above, the GrimAge clock includes a component DNAm surrogate for lifetime smoking exposure. Therefore, tobacco use is a fundamental part of the estimate of GrimAA. Adjusting for tobacco use, then, to some extent undermines the design of the GrimAge clock, while not adjusting for tobacco use limits the interpretation of associations with GrimAA. At the center of this conundrum is a concept introduced earlier: that GrimAge is a tool derived as a product of research with predictive aims, but that researchers employ in work with causal inference aims. In order to build an excellent predictive tool, the GrimAge clock includes components that risk confounding in causal work, and cloud the distinction between total, direct, and indirect causal effects. To add further complexity, the risks of not adjusting for tobacco exposure depend on whether smoking is felt to be a confounder or a mediator of the association of stress and psychopathology with GrimAA [55]. In Fig. 2, we have depicted smoking as a mediator, where stress/psychopathology causes tobacco use, and tobacco use in turn causes GrimAA. Therefore the decision to adjust for smoking in this model is a decision to calculate either a total or a direct causal effect. However, it is possible that the relationship of stress/psychopathology with tobacco use is instead a confounded one, wherein genetic and environmental factors predispose individuals to both tobacco use and stress/psychopathology [55]. If this is the case, then adjustment for tobacco exposure is essential to yield unconfounded estimates of association between stress/psychopathology and GrimAA (see Supplementary Fig. 2). Most of the studies reviewed here acknowledge this complexity and address it by statistical adjustment for tobacco use. Some studies have incorporated additional steps. For instance, both Protsenko et al. and Yang et al. calculate an additional GrimAA metric that excludes the DNAm surrogate for tobacco exposure. In Protsenko et al., we also took the additional step of completing sensitivity analyses that restricted the sample on the basis of smoking history. So far, there is no gold-standard approach for how to handle tobacco exposure in GrimAge analysis.

A more challenging question is whether and how to address the fourth suspected mediator: comorbid somatic illness. The mechanisms by which stress and psychopathology cause medical illness — such as autonomic, neuroendocrine, and immune changes [52–54] — are likely similar to the mechanisms by which they contribute to epigenetic aging. Theoretically, adjusting for somatic illness would obscure the effect of interest, and therefore would be unnecessary and undesirable. By this same rationale, we might expect to see an attenuation of the association between stress and GrimAA in studies that account for somatic disease. Yet, in the studies reviewed here, we observe a trend towards the reverse: the three studies that restricted their samples based on somatic health all reported significant results [28, 30, 33], while the remaining studies with no health-related exclusions had mixed findings. One possible explanation may be the strong association between somatic disease and GrimAA. GrimAge’s distinguishing feature among the epigenetic clocks is its ability to predict morbidity and mortality. It is possible that the magnitude of effect of somatic disease on GrimAA is so much larger than that of stress or psychopathology that, when studied in the general population, the signal of interest cannot be detected. Indeed, effect sizes for the association between stress and psychopathology with GrimAA were generally modest in the studies reviewed here, based both on the magnitude of excess GrimAA observed and on standardized measures of effect size like Cohen’s *d*. (There are other scenarios that might explain why studies restricted to somatically healthy adults may be more likely to report significant results, e.g., selection bias. The depiction of selection bias in DAGs is detailed in the Supplementary Materials and Supplementary Fig. 3).

One final consideration is about mediating role of blood cell composition. It is well documented that the second-generation clocks correlate with blood cell composition [4, 9, 10], and the DNAm Age calculators output Houseman estimates of composition [42]. It is not clear how stress and psychopathology may affect blood cell composition [54, 56], and this is perhaps another mechanism by which stress and psychopathology contribute to premature morbidity and mortality. If so, this issue highlights that the choice of covariates is inextricably linked to the precise research question at hand. If researchers are interested in estimating the total effect of all biological mechanisms by which stress and psychopathology contribute to GrimAA, then adjusting for blood cell composition would remove meaningful signal. Similarly, researchers might be specifically interested in complex pathways that are mediated by blood cell composition, making blood cell composition the signal of primary interest. On the other hand, it is also important to identify other biological mechanisms independent of the well-known association of GrimAge with blood cell composition. Therefore, for research questions not specifically interrogating mechanisms mediated by blood cell composition, it is generally beneficial to report results both with and without adjustment for blood cell composition.

The remaining variables depicted in Fig. 2 are confounders of the association of stress and stress-related psychopathology with GrimAA. It should be noted that adequate control of confounding does not require adjustment for all variables. Rather, when we shift focus from individual covariates to the complex system of variables surrounding the research question, the analytic approach to confounding becomes a strategic effort to close “confounding paths”. Often, confounding. paths can be closed by adjusting for only a single variable along the path. To identify the necessary covariates, online tools such as dagitty.net [57] provide a “minimally sufficient adjustment set” (MSAS) based on a DAG. The MSAS for estimation of the direct effect of stress/psychopathology on GrimAA includes only Race + Sex + Adult SES as confounders. While controlling for sex was nearly universal in the studies reviewed here, SES and race were not consistently accounted for. While some of the effects of SES are mediated by behavioral variables, further adjustment is still necessary if it is felt that there are additional mechanisms by which SES alters GrimAA (e.g., environmental exposures). Meanwhile, race, ethnicity, and/or genetic ancestry are considered only in three studies [29–31]. There has been concern raised that at least one of the epigenetic clocks (PhenoAge) may exhibit racial bias [58], and another study found disparities in GrimAA between Black and White samples [59]. Therefore, adjustment may be warranted. However, because the epigenetic changes underlying GrimAA are hypothesized to be in part a function of lived experience, adjustment for race and ethnicity may attenuate signal due to differences in the burden of race-related trauma, rather than race per se. Interestingly, McKenna et al. examined the associations between race-related lifetime stress and GrimAA in a sample of Black participants, and found no associations, although they did find evidence of an indirect effect mediated by internalized anger expression. Without a larger body of literature specifically addressing the relationship of race to GrimAA, adjustment may be a useful sensitivity analysis.

Based on this review, we offer the following recommendations for future work with epigenetic clocks:

- Construct a directed acyclic graph (DAG) depicting the complex system of variables surrounding the association of interest. Including this DAG in the published work — either as a primary or supplementary figure — will make clear to readers how the authors conceptualize the research question, and support consensus theory and methods. (For an introduction to constructing DAGs, we recommend http://dagitty.net/learn/, Digitale et al. [44] and Shrier and Platt [60]).
- Explicitly state the research question in terms of total, direct, and indirect causal effects.
- Select covariates on theoretical grounds, based on the DAG. Use tools such as dagitty.net to identify variables that must be adjusted for to eliminate confounding, and mediators that must be adjusted for to calculate direct rather than total effects.
- When there is uncertainty as to the role or relevance of a covariate, report serially adjusted models. While covariates should be selected a priori on theoretical grounds, we recommend that authors provide data on the associations between the covariates considered and GrimAA. DAGs are evolving systems that grow with our increasing knowledge. Providing such information will help to define correct DAGs in the future and advance best practices in the field.

This review has several limitations. First, we focused only on the GrimAge clock and did not review associations of stress and stress-related psychopathology with the other epigenetic clocks, despite a robust literature on this topic. Indeed, several of the papers reviewed here assessed multiple epigenetic clocks, and at times found significant associations with other clocks but not with GrimAge. However, our focus was on applying principles from the field of epidemiology to the study of epigenetic age, and a limited focus facilitated this discussion. We chose GrimAge due to the unique challenges that this clock presents, and because of the field’s increasing interest in the GrimAge clock. Additionally, this is not an exhaustive review of all factors that may contribute to heterogeneity of results. For instance, the studies of PTSD reviewed here differed in the time between exposure to trauma and measurement of GrimAA. Additional studies are needed to understand the timescale over which epigenetic age acceleration occurs following exposure to stress or onset of psychopathology, and such work will inform future study design. Finally, future work may show that stress, PTSD, and MDD are all heterogeneous entities, and their subtypes may differ in their relationship with epigenetic age.

In summary, we have reviewed the literature on associations of stress and stress-related psychopathology with GrimAA. While mixed results and varied methods preclude a clear conclusion, the body of work presented suggests that accelerated epigenetic aging is a promising avenue of research in stress, PTSD, and MDD. We suggest that the methods employed in clinical epidemiology will advance future work by using DAGs to define the “universe” of covariates within which the research question exists and building consensus on optimal analytic methods. Future work built on this understanding will (1) use DAGs as a means to clearly communicate the research question and analytic rationale, (2) refine the research question in terms of total, direct, and indirect causal effects to better reflect our interest in the varied biological and behavioral mechanisms involved, (3) disentangle methodological differences from true biological differences, and perhaps most importantly, (4) help us to understand the implications of epigenetic aging for psychiatric care. Despite the long-held idea that psychological stress contributes to aging, the field of accelerated biological aging in psychiatric illness is still young. Ultimately, understanding this relationship will require long-term prospective research. While we await such studies, we hope that the framework outlined in this review will facilitate high-quality cross-sectional research built on consensus methods and with important implications for psychiatric care.

## Supplementary information

Supplemental Material
