# Supplementary material for: Associations of stress and stress-related psychiatric disorders with GrimAge acceleration: review and suggestions for future work
Source: Transl Psychiatry. 2023 May 2;13:142. doi: 10.1038/s41398-023-02360-2 (PMC10154294; doi:10.1038/s41398-023-02360-2)
Supplement: Supplementary file 1 — Supplemental Material [file 41398_2023_2360_MOESM1_ESM.pdf]

Supplementary Materials

Code for DAG in dagitty.net:

The following model code may be input to the 'model code' panel at dagitty.net to reconstruct the DAG in **Figure 1**:

```
dag {
"Adult SES" [pos="-1.297,-1.095"]
"Alcohol Use" [pos="-0.482,-0.970"]
"Blood Cell Composition" [pos="1.003,-0.673"]
"Childhood SES" [pos="-1.649,-0.504"]
"Medical Illness" [pos="0.620,-1.135"]
"Physical Activity" [pos="-0.505,-1.610"]
"Selection Into Study" [pos="1.211,-2.130"]
"Stress & Stress-Related Psychopathology" [exposure,pos="-1.370,0.675"]
"Tobacco Use" [pos="-0.502,-1.300"]
BMI [pos="-0.490,-0.684"]
Education [pos="-1.937,-1.085"]
GrimAA [outcome,pos="0.710,0.660"]
Race [pos="-2.202,-0.504"]
Sex [pos="-2.171,0.166"]
"Adult SES" -> "Alcohol Use"
"Adult SES" -> "Medical Illness"
"Adult SES" -> "Physical Activity"
"Adult SES" -> "Stress & Stress-Related Psychopathology"
"Adult SES" -> "Tobacco Use"
"Adult SES" -> BMI
"Adult SES" -> GrimAA
"Alcohol Use" -> "Medical Illness"
"Alcohol Use" -> GrimAA
"Blood Cell Composition" -> GrimAA [pos="1.086,0.735"]
"Childhood SES" -> "Adult SES"
"Childhood SES" -> "Stress & Stress-Related Psychopathology"
"Childhood SES" -> Education
"Medical Illness" -> "Blood Cell Composition" [pos="1.045,-1.165"]
"Medical Illness" -> GrimAA
"Physical Activity" -> "Medical Illness"
"Physical Activity" -> GrimAA
"Stress & Stress-Related Psychopathology" -> "Alcohol Use" [pos="-0.992,-0.434"]
"Stress & Stress-Related Psychopathology" -> "Blood Cell Composition" [pos="0.412,-0.584"]
"Stress & Stress-Related Psychopathology" -> "Medical Illness" [pos="-0.513,-0.424"]
"Stress & Stress-Related Psychopathology" -> "Physical Activity" [pos="-1.213,-0.839"]
"Stress & Stress-Related Psychopathology" -> "Tobacco Use" [pos="-1.083,-0.654"]
"Stress & Stress-Related Psychopathology" -> BMI [pos="-0.889,-0.384"]
"Stress & Stress-Related Psychopathology" -> GrimAA
"Tobacco Use" -> "Medical Illness"
"Tobacco Use" -> GrimAA
BMI -> "Medical Illness"
BMI -> GrimAA
Education -> "Adult SES"
Race -> "Adult SES"
Race -> "Childhood SES"
Race -> "Stress & Stress-Related Psychopathology"
Race -> GrimAA
Sex -> "Stress & Stress-Related Psychopathology"
Sex -> GrimAA
}
```

Supplementary Figure 1:

Supplementary Figure 1: Step-by-Step Construction of the Directed Acyclic Graph in Figure 2. All images were created using dagitty.net and modified for clarity.

(see next page)

(A)

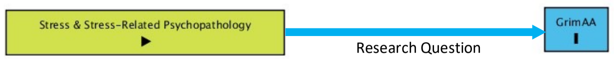

Directed acyclic graphs (DAGs) depict the "universe" of covariates surrounding a research question. They are constructed *a priori* to aid in appropriate covariate selection for causal inference. In the first step, we define the research question (RQ). Here, the RQ is "do stress and stress-related psychopathology cause acceleration of the GrimAge clock (GrimAA)?"

(B)

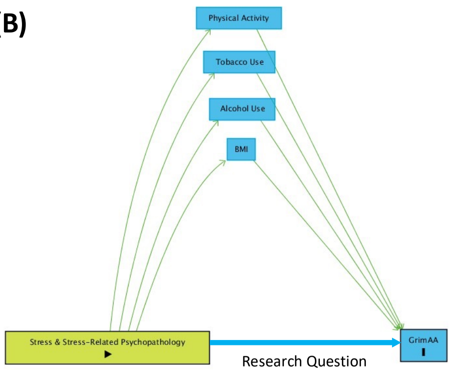

In the next step, we depict the behavioral mediators, based on the Oblak et al., 2021 review. This list of behavioral mediators is not intended to be exhaustive, but rather is a practical starting point based on current literature. Of note, the direction of the depicted relationships remains controversial, and some of variables may instead be confounders. See Supplementary Materials for more.

(C)

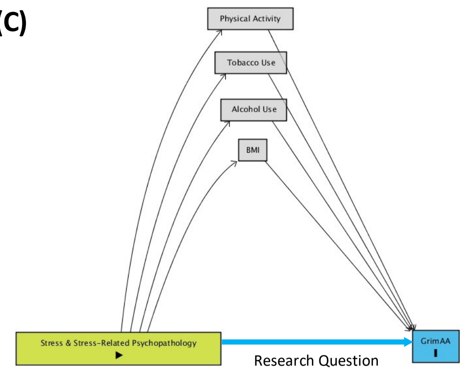

In this simplified DAG, the distinction between total (all arrows), direct (blue arrow) and indirect (grey arrows) causal effects is made clear. However, because stress and psycho-pathology are complex exposures, researchers must still define what is meant by "direct" and "indirect" effects. Here, we consider effects mediated by known health-related behaviors to be indirect, while biological mechanisms are direct.

(D)

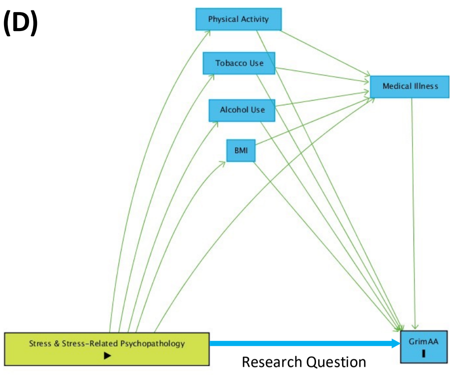

Comorbid medical illness is a challenging variable to address in analysis of stress/psycho-pathology and GrimAA. We depict it as a mediator, with suspected mechanisms including autonomic, neuroendocrine, and immune changes. However, medical illness is also a risk factor for stress and psychopathology, and therefore could be depicted as a confounder.

(E)

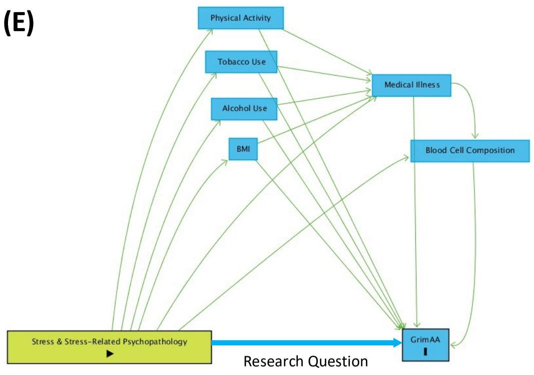

Studies of GrimAA commonly adjust for blood cell composition because the 2nd –generation clocks are associated with changes in composition. However, changes in blood cell composition may also be a mechanism of clinical and scientific interest. Whether or not to adjust for blood cell composition depends entirely on the precise research question. Generally, providing both unadjusted and adjusted estimates of causal effect will be valuable.

(F)

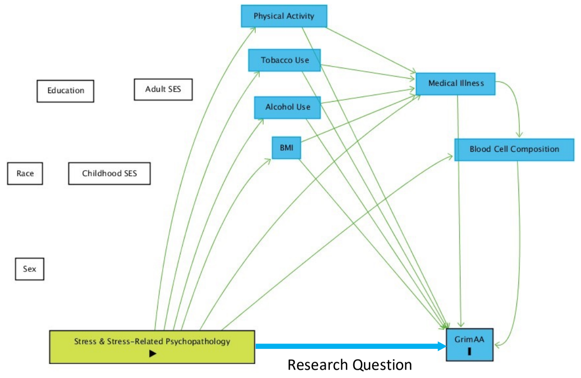

In the next step, we consider the complex web of confounders that influence stress, risk of psychopathology, GrimAA, and the behavioral intermediates. For simplicity, here we show the five confounders considered without their relationships to other variables. There are also many other variables that could considered, such as environmental exposures and adverse childhood experiences.

(G)

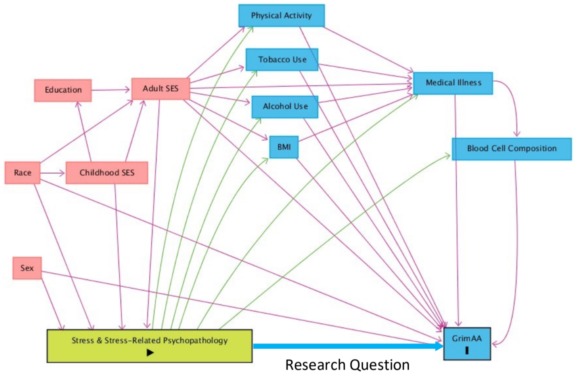

As we add the causal relationships of the confounders to other variables, the DAG becomes complex. The mediators are now also part of confounding paths, as indicated by the pink rather than green lines. As DAG complexity increases, it becomes difficult to determine the covariates that must be adjusted for. To address this, dagitty.net provides a "minimally sufficient adjustment set" (MSAS) for both total and direct causal effects. A single DAG can have multiple MSAS options, and an MSAS can be selected based on the available data, most practical variables to measure, etc.

(H)

POSSIBLE FUTURE ADDITIONS

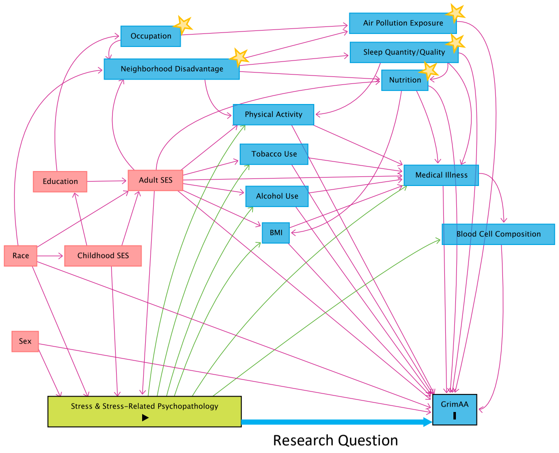

DAGs are evolving theory-based representations of a causal system, where the variables included and their relationships with one another change with new data and understanding. NOTE that this figure is hypothetical and is not intended to depict the current state of the field.

Adjusting for Smoking Exposure

As noted in the main text, we depict smoking exposure as a mediator of the association between stress and stress-related psychopathology and GrimAA. As detailed in Ziedonis et al., the causal link of PTSD and MDD with smoking could be due to changes in HPA axis function following stress and trauma exposure, attempts to regulate negative emotional states associated with anxiety disorders, altered reward processing related to smoking, and increased vulnerability to social pressures related to smoking, among other mechanisms[1]. However, it is also possible that smoking is a confounder of the relationship between stress and psychopathology and GrimAA. In this model, smoking exposure could either directly increase the risk of stress and stress-related psychopathology – perhaps through changes in neurophysiology – or some other genetic or environmental factor predisposes to both stress/psychopathology and tobacco use. Both of these scenarios are depicted in Supplementary Figure 1.

Of note, both scenarios require adjustment for tobacco use, but for different reasons. In the scenario where tobacco use is a mediator, adjustment is required only to yield a direct causal effect estimate. This was detailed in the main text. In the scenario where tobacco use is a confounder, adjustment is necessary to yield any valid estimate of causal effect, including total causal effect.

Supplementary Figure 2:

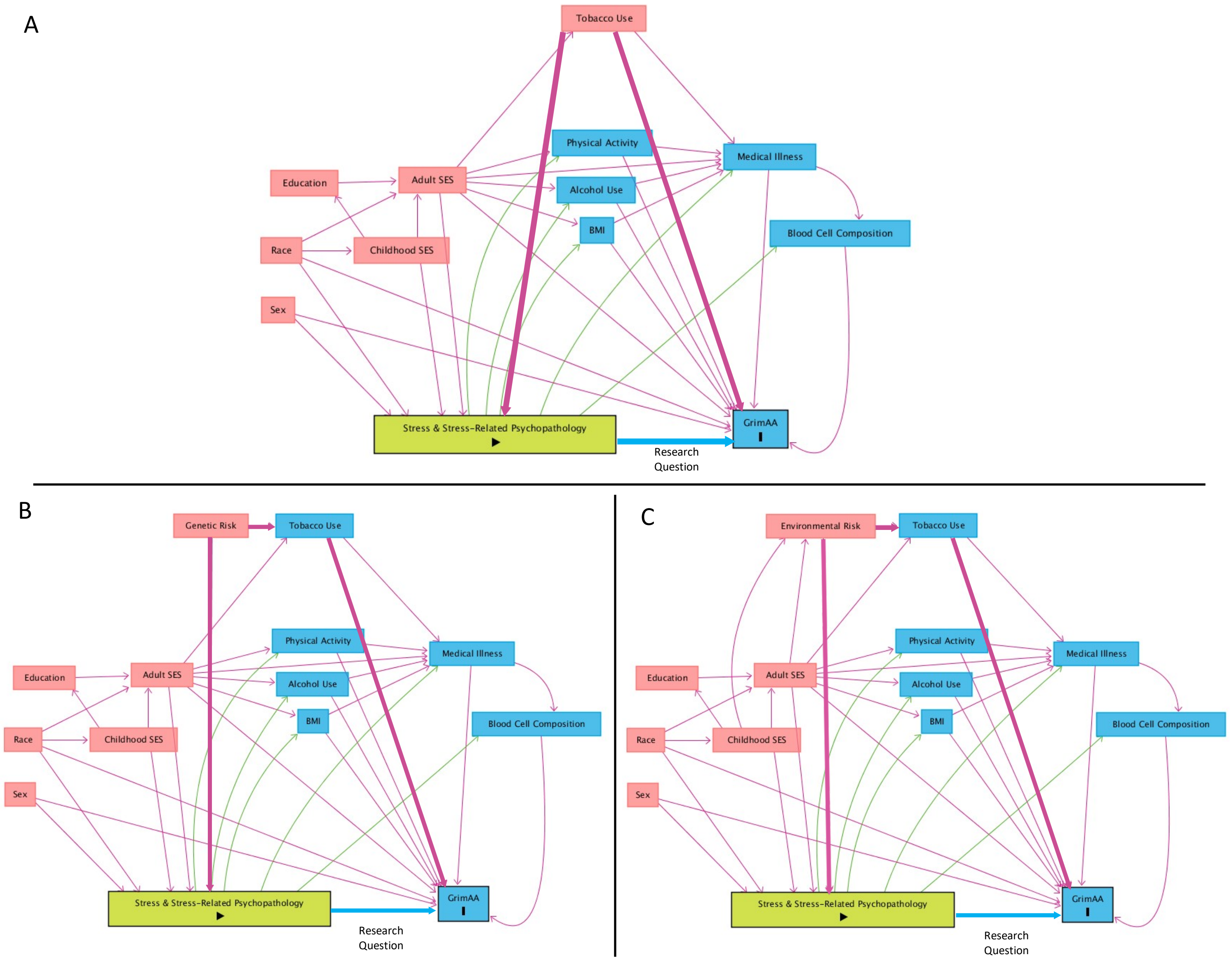

Supplementary Figure 2: Directed Acyclic Graph Depicting Tobacco Use as a Confounder. A. Tobacco may be a confounder of the association between Stress & Stress-Related Psychopathology and GrimAA by directly contributing to risk of stress and stress-related psychopathology. Adjustment for tobacco use will close the confounding path. **B.** Tobacco use may be part of a confounding path caused by shared genetic risk factors for stress/stress-related psychopathology and tobacco use. Adjusting for either tobacco use or genetic risk will close the confounding path. **C.** Tobacco use may be part of a confounding path caused environmental risk factors for both stress/stress-related psychopathology and tobacco use. Again, adjusting for either tobacco use or environmental risk will close the confounding path. However, tobacco use is more practically quantifiable than either genetic or environmental risk.

On Comorbid Somatic Illness - Selection Bias and “Adjusting for a Collider”:

In a DAG, a “collider” is any variable that is a common effect of the exposure and the outcome. When colliders are adjusted for, they can induce a spurious statistical association between the exposure and outcome when no true causal association exists. In other words, controlling for a collider by statistical adjustment, stratification, or sample restriction introduces bias. Identifying colliders and their impacts on statistical associations is perhaps the most complex foundational principle in DAG use. They are easiest to identify when the collider is directly caused by both the exposure and the outcome. However, collider stratification bias can also occur when the collider and the exposure and/or outcome are associated by other means (e.g., a fourth variable causes both the collider and the exposure, and a fifth variable causes both the collider and the outcome). Several excellent resources are available for further information on colliders. See Cole et al., 2010[2] and Shrier & Platt, 2008[3] for an introduction, and Hernan et al., 2004[4] for more detail. Online tools such as dagitty.net can identify collider stratification bias if the collider is included as a node in the DAG and identified as “adjusted”.

Importantly, “collider stratification bias” is the DAG representation of a much more familiar concept – selection bias[4]. Selection bias occurs anytime that selection into a study is related to both the exposure and the outcome of interest. “Selection into the study” is the collider in this case, and is by necessity stratified upon when we analyze only the subset of the target population who enrolled in the study. Several biomedical “paradoxes” have been attributed to inadvertent collider stratification due to selection bias (e.g., obesity paradox[5]).

In the studies reviewed here, selection bias can arise from several sources (**Supplementary Figure 1**). In studies that employed exposure-selective sampling (e.g., PTSD patients and PTSD-free controls), we can imagine that incentives to volunteer for a study may differ between participants with and without PTSD. For instance, those with PTSD might be highly motivated to enroll in a study to contribute to science despite other barriers to participation, such as medical illness or low socioeconomic status. Meanwhile, PTSD-free controls might lack the same intrinsic motivation to participate, and those who do may be more likely to be somatically well and able to attend a study. However, it may be difficult to predict how patients and controls may differ and in which direction the bias is likely to be.

Supplementary Figure 3:

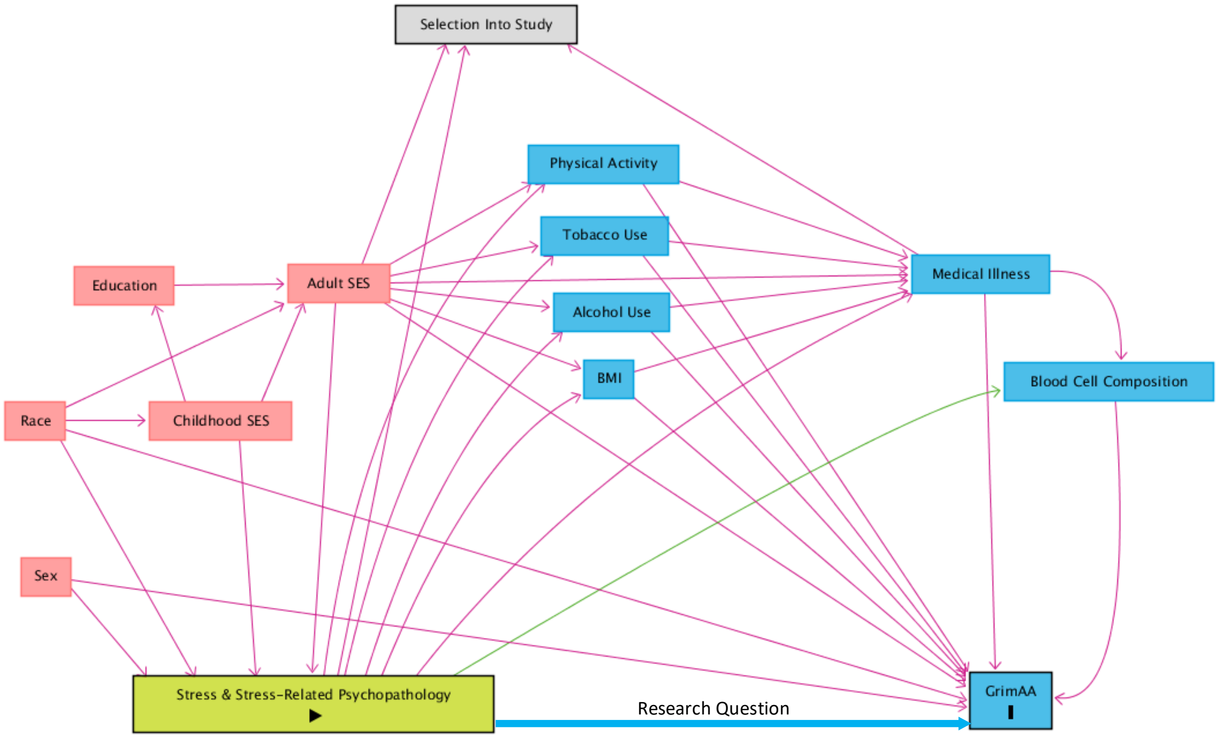

Supplementary Figure 3: Directed Acyclic Graph Depicting Collider Stratification Bias. A collider is any variable that is a common effect of both the exposure and the outcome. Variables can also be colliders when there is another variable that is a common cause of both the exposure and the collider, or the outcome and collider. In this model, 'Medical Illness' is a common cause of both 'Selection into Study' and 'GrimAA,' while 'Adult SES' is a common cause of both 'Stress & Stress-Related Psychopathology' and 'Selection into Study.' When a collider is adjusted for, it introduces bias into the analysis. In the case of selection bias, 'Selection into Study' is conditioned upon because investigators are only able to study the stratum of individuals who enrolled in the study and for whom data was collected. This type of selection bias is often difficult to detect without a DAG, and maybe be a challenge for observational studies relying on research volunteers.

	Comparison	Unexposed: Mean GrimAA (SD) (Years) N	Exposed: Mean GrimAA (SD) (Years) N	Cohen's D (Unadjusted Models)
Katrinli et al., 2020	No PTSD vs. Current PTSD	Control: -0.37 (4.85) N = 427	Current PTSD: 0.47 (4.82) N = 218	0.17
	No PTSD vs. Lifetime PTSD		Lifetime PTSD: 0.41 (4.91) N = 209	0.16
Yang et al., 2021	Control vs. PTSD (Discovery Cohort)	Control: -0.57 (3.38) N = 82	PTSD: 1.26 (3.93) N = 80	0.50
	Control vs. PTSD (Validation Cohort)	Control: -1.60 (2.96) N = 27	PTSD: 0.93 (3.73) N = 26	0.75
Wang et al., 2021	No PTSD vs. PTSD	No Current PTSD: -0.13 (5.50) N = 272	PTSD: 1.49 (4.93) N = 24	0.31 (not sig)
Protsenko et al., 2021	Control vs. MDD	Control: -0.29 (1.01) N = 60	MDD: 0.30 (0.84) N = 49	0.63

Supplementary Table 1: Cohen's d Effect Sizes for Unadjusted Comparisons of either PTSD to Control Participants or MDD to Control Participants. Effect sizes were calculated for all studies providing sufficient data to do so.

Supplementary References

1. Ziedonis, D. *et al.* Tobacco use and cessation in psychiatric disorders: National Institute of Mental Health report. *Nicotine Tob. Res.* **10**, 1691–1715 (2008).
2. Cole, S. R. *et al.* Illustrating bias due to conditioning on a collider. *Int. J. Epidemiol.* **39**, 417–420 (2010).
3. Shrier, I. & Platt, R. W. Reducing bias through directed acyclic graphs. *BMC Med. Res. Methodol.* **8**, 70 (2008).
4. Hernán, M. A., Hernández-Díaz, S. & Robins, J. M. A Structural Approach to Selection Bias: *Epidemiology* **15**, 615–625 (2004).
5. Lajous, M., Banack, H. R., Kaufman, J. S. & Hernán, M. A. Should Patients with Chronic Disease Be Told to Gain Weight? The Obesity Paradox and Selection Bias. *Am. J. Med.* **128**, 334–336 (2015).
